# Brain structure correlates of emotion-based rash impulsivity

**DOI:** 10.1016/j.neuroimage.2015.04.061

**Published:** 2015-07-15

**Authors:** N. Muhlert, A.D. Lawrence

**Affiliations:** School of Psychology and Cardiff University Brain Research Imaging Centre (CUBRIC), Cardiff University, Cardiff, UK

**Keywords:** Emotion regulation, Grey matter volume, Impulsivity, Personality, Urgency, Voxel-based morphometry

## Abstract

Negative urgency (the tendency to engage in rash, ill-considered action in response to intense negative emotions), is a personality trait that has been linked to problematic involvement in several risky and impulsive behaviours, and to various forms of disinhibitory psychopathology, but its neurobiological correlates are poorly understood. Here, we explored whether inter-individual variation in levels of trait negative urgency was associated with inter-individual variation in regional grey matter volumes. Using voxel-based morphometry (VBM) in a sample (n = 152) of healthy participants, we found that smaller volumes of the dorsomedial prefrontal cortex and right temporal pole, regions previously linked to emotion appraisal, emotion regulation and emotion-based decision-making, were associated with higher levels of trait negative urgency. When controlling for other impulsivity linked personality traits (sensation seeking, lack of planning/perseverance) and negative emotionality per se (neuroticism), these associations remained, and an additional relationship was found between higher levels of trait negative urgency and smaller volumes of the left ventral striatum. This latter finding mirrors recent VBM findings in an animal model of impulsivity. Our findings offer novel insight into the brain structure correlates of one key source of inter-individual differences in impulsivity.

## Highlights

• We identified brain structure correlates of rash impulsivity (negative urgency).
• Urgency was inversely linked to dorsomedial frontal & right temporal pole volumes.
• Controlling for neuroticism, urgency also linked to lower ventral striatal volumes.

## Introduction

The personality trait of “impulsivity” has long been implicated as an important risk and maintaining factor across a diverse range of risky behaviours and mental health problems (DeYoung et al., 2010; Dalley et al., 2011). It is increasingly apparent, however, that there is no single impulsivity trait. Rather, there are separate personality traits underlying various forms of impulsive behaviour (Birkley and Smith, 2011; Sharma et al., 2013). According to one influential model, the UPPS model (Whiteside and Lynam, 2001), the construct of impulsivity encompasses four largely distinct “impulsigenic” (Sharma et al., 2013) traits or dispositions: sensation seeking (the tendency to seek excitement), lack of planning (the tendency not to plan or think ahead), lack of perseverance (the inability to sustain attention and motivation on boring tasks) and urgency (the tendency to act rashly when distressed). Subsequent revisions to this model (Birkley and Smith, 2011; Cyders and Smith, 2008; Sharma et al., 2013) have revealed that urgency has two facets (negative and positive urgency, referring to the tendency to act rashly when experiencing intensively negative or positive mood states, respectively) and that lack of planning and lack of perseverance represent two facets of a lack of conscientiousness.

A notable feature of the UPPS model is that it has drawn attention to emotion-related impulsigenic traits that were largely neglected previously. In particular, one facet of impulsivity, negative urgency, refers specifically to the disposition to act rashly in the context of intense negative emotional states (Whiteside and Lynam, 2001; Cyders and Smith, 2008). After controlling for the other impulsivity-related traits (sensation-seeking, lack of planning/ perseverance) and for the tendency to experience negative emotions per se, negative urgency has been shown, across multiple populations, including adolescent, undergraduate, adult community and clinical samples, to be a uniquely important cross-sectional and prospective predictor of problematic involvement in several maladaptive and risky behaviours, including susceptibility to negative peer influence, risky sexual behaviour, reckless driving, intimate partner violence, smoking, alcohol problems and illegal drug use (Cyders and Coskunpinar, 2010; Dir et al., 2013; Settles et al., 2012; Stautz and Cooper, 2014). Additionally, negative urgency is the impulsigenic trait most strongly linked to the severity of psychopathology in clinical psychiatric disorders such as binge eating disorder (Racine et al., 2013), pathological gambling (Michalczuk et al., 2011) and borderline personality disorder (Whiteside et al., 2005). In particular, negative urgency is an important personality contributor across the spectrum of so-called externalizing or disinhibitory disorders (e.g. alcohol dependence, drug use, aggression and conduct disorders) (Settles et al., 2012), and may represent the personality “core” of the externalizing disorders (Clark, 2005). A critical issue for impulsivity neuroscience, then, is to determine the neurobiological underpinnings of the negative urgency trait.

Recent structural magnetic resonance imaging (MRI) studies have found that inter-individual variation in a number of behavioural traits – including personality traits – can be predicted from the local structure of grey matter as assessed by voxel-based morphometry (VBM) (Kanai and Rees, 2011). Here, we used VBM to examine the relationship between trait negative urgency and local grey matter volumes in a sample of healthy individuals. Negative urgency can be considered to represent a personality process by which subjective distress leads to disagreeable, ill-considered rash action, and shares variance with the ‘big five’ personality traits of high neuroticism, low conscientiousness and low agreeableness (Cyders and Smith, 2008; Settles et al., 2012). DeYoung et al. (2010), using brain structure morphometry, found that lower local volumes of medial/superior frontal gyrus (dorsomedial prefrontal cortex) were linked to high neuroticism, but also to low agreeableness and to low conscientiousness. Relatedly, negative urgency is thought to reflect, in part, underlying problems with emotion regulation (Carver et al., 2008; Cyders and Smith, 2008) and activity in the dorsomedial prefrontal cortex (dmPFC) is associated with both instructed and spontaneous emotion regulation (Ochsner et al., 2004; Silvers et al., 2014a). Indeed, the dmPFC has been shown in a recent fMRI meta-analysis to be the region most consistently active across 48 studies of emotion regulation (Buhle et al., 2013). Hence, we predicted that, across individuals, higher levels of trait negative urgency would be associated with smaller local grey matter volume in the dmPFC. In addition, individuals with co-occurring cocaine dependence and personality disorder, characterised by very high levels of trait negative urgency, have recently been shown to have smaller grey matter volume of the right temporal pole (Albein-Urios et al., 2013). We therefore also examined potential associations between higher levels of negative urgency and smaller grey matter volumes in right temporal polar regions. To examine the unique variance explained by negative urgency, we additionally controlled for individual differences in the other impulsivity-related traits of the UPPS model (sensation seeking, lack of planning/ perseverance) and negative emotionality per se (i.e. trait neuroticism). Our findings provide novel insight into the neurobiological variations that reflect inter-individual differences in tendencies towards rash, impulsive behaviours in the context of heightened negative emotions.

## Methods

### Sample

One-hundred and fifty-two healthy right-handed participants, primarily undergraduate and graduate university students (109 females; mean age: 23.6 years [standard deviation: 5.4 years]), underwent structural magnetic resonance imaging (MRI) after providing written informed consent and screening for MRI contra-indications, including a history of neurological or psychiatric disorders. The study was approved by the Cardiff University School of Psychology Research Ethics Committee.

### Negative urgency

Trait negative urgency was measured using the UPPS Impulsive Behaviour Scale (Whiteside and Lynam, 2001), a 45-item scale formed from a factor analysis of a wide-range of self-report impulsivity scales, and designed to assess urgency, lack of planning, lack of perseverance and sensation seeking. The 12-item negative urgency subscale contains questions such as “I am always able to keep my feelings under control” and uses a 4-point Likert-type scale ranging from 1 (*agree strongly*) to 4 (*disagree strongly*). Higher mean scores reflect higher levels of impulsivity. It has excellent internal consistency, with Cronbach's alpha level typically exceeding 0.8 (in this study, Cronbach's alpha was 0.79). An extensive body of evidence supports the validity of the negative urgency scale. The scale has repeatedly emerged as uni-dimensional, scores converge across self-report and interview assessment methods (Smith et al., 2007), and the scale is gender invariant (i.e. measures the same trait in women and men) (Cyders, 2013). Further, trait negative urgency has excellent discriminant validity in comparison to measures of the other UPPS traits, and correlates with criterion variables as predicted by theory (for a review, see Birkley and Smith, 2011; Cyders and Smith, 2008).

Here, mean scores (out of a maximum possible score of four) for negative urgency were calculated and used in all analyses. The other traits from the UPPS (lack of planning, lack of perseveration and sensation-seeking) were also scored and used as covariates.

### Negative emotionality (neuroticism)

Trait neuroticism (the tendency to experience negative emotions) was measured using the neuroticism scale of the Big Five Inventory (BFI) 44-item version (John et al., 2008). This self-report personality scale was completed by a subset of 139 of the 152 participants described above (109 female, mean age: 23.2 years, standard deviation: 5.3). The neuroticism scale was not acquired in 13 subjects for logistical reasons. The BFI-44 neuroticism scale features eight short-phrase items, such as “I see myself as someone who is depressed, blue”, rated on a 5-point Likert-type scale ranging from 1 (*disagree strongly*) to 5 (*agree strongly*). In this study, Cronbach's alpha for Neuroticism was 0.85, indicating excellent internal consistency.

### MRI data-acquisition and pre-processing

MRI scans were obtained on a 3 T GE HDx signa MRI scanner at the Cardiff University Brain Research Imaging Centre (CUBRIC) fitted with an 8-channel head coil. High resolution T1-weighted fast spoiled gradient (FSPGR) coronal scans (TR = 7.9 ms, TE = 3 ms, inversion time = 450 ms, flip angle = 20^0^, 1 mm isotropic resolution) were acquired with 168 or 172 slices depending on head size.

Images were segmented using SPM8 (Wellcome Trust Centre for Neuroimaging, London) into grey matter, white matter and cerebrospinal fluid tissue classes using unified segmentation. Grey matter images were then normalised to a grey matter population template, generated from the complete image set using the diffeomorphic anatomical registration using exponentiated lie-algebra (DARTEL) registration method (Ashburner, 2007). This nonlinear warping technique minimises between-subject structural variations. All images were checked following segmentation and normalisation to ensure the accuracy of these steps. The final voxel resolution was 1 × 1 × 1 mm^3^. Spatially normalised images were modulated by the Jacobian determinants so that intensities represent the amount of deformation needed to normalise the images, and then smoothed with an 8-mm full-width at half-maximum Gaussian kernel.

### Voxel based morphometry: statistical analysis

Voxel-based multiple regression analysis (based on the general linear model: GLM) was carried out using SPM8 with voxel-wise grey matter volume (GMV) as the dependent variable. Age and gender (known predictors of brain volume) were added as nuisance covariates to the GLM (Barnes et al., 2010). Negative urgency mean score was used as the predictor. Total intracranial volumes were calculated by summing the values of the native space tissue segmentations (grey matter, white matter and cerebrospinal fluid) using the ‘get_totals’ function in SPM8 and added as a global measure for proportional global scaling (Peelle et al., 2012). Resulting SPM t-maps were superimposed on the MNI single subject brain and labelling was carried out using the AAL atlas (Tzourio-Mazoyer et al., 2002) included in the MRIcron software package (http://www.mccauslandcenter.sc.edu/mricro/mricron/). Results were visualized using NeuroElf (www.neuroelf.net).

Statistical analysis was then carried out in the following stages.

1. Correlations between negative urgency scores and grey matter volumes were assessed using a region of interest (ROI) based approach, with ROIs sampled using 10 mm diameter spheres (drawn using the WFU_PickAtlas, Maldjian et al. (2003)) centred on previously identified peak voxels in the following regions (see introduction):
  a. Associations with grey matter volume in the dmPFC (MNI coordinates: 3, 38, 39), based on the VBM study of personality by DeYoung et al. (2010).
  b. Associations with grey matter volume in the right temporal pole (48, 11, − 30), based on lower levels of volumes in this region reported in individuals with co-occurring cocaine dependence and personality disorder (Albein-Urios et al., 2013).
  In each ROI, associations were corrected for multiple comparisons using Family Wise Error (FWE) correction based on Gaussian Random Field Theory (RFT) (p < .05, small volume corrected for a priori ROI). We then looked for any other regions throughout the brain that showed associations with negative urgency (p < .001, corrected for multiple comparisons based on cluster extent, accounting for non-isotropic smoothness [456 voxels]). To test for potential sex differences in the association between regional grey matter volume and negative urgency, we additionally formally tested for an interaction between gender and negative urgency scores in SPM using a full factorial model (which accounts for unequal variance) with the factor of gender, the covariates of negative urgency and age and the global factor of intracranial volume for proportional global scaling.
2. To assess the amount of unique variance captured by negative urgency, we repeated the regression after including mean [lack of perseveration and lack of planning] and sensation-seeking (as well as age and gender) as nuisance covariates. An averaged score combining (lack of) perseveration and (lack of) planning was used due to the high correlation between these two measures (r = .51, p < .001), consistent with previous findings that (lack of) perseveration and (lack of) planning are correlated facets of (low) conscientiousness (Birkley and Smith, 2011).
3. We then repeated the ROI analyses from (1) in the subset of participants (n = 139, see above) who had completed both the UPPS and the BFI-44 neuroticism scales, after including mean neuroticism score from the BFI-44 as a covariate of no interest. This analysis allowed us to examine the relation between regional grey matter volume and levels of emotion-driven rash impulsivity, controlling for inter-individual differences in the tendency to experience negative emotions per se.

## Results

Mean scores on the UPPS traits, including negative urgency, together with mean levels of trait neuroticism are reported in Table 1. There was a broad range in reported trait negative urgency, with individual mean scores ranging from 1.08 to 3.58, spanning almost the entire range of possible scores (1–4). Mean levels of trait negative urgency (2.1) in our sample were comparable to those reported in a large (n > 1300) sample of undergraduate students from the USA (Cyders, 2013) (mean score = 2.3). Here, there was a non-significant trend for women to report higher levels of negative urgency than men (t = 1.9, p = 0.06, Cohen's d = 0.3). Larger samples (e.g. Cyders, 2013) report no sex differences in mean levels of negative urgency.

### ROI-based analysis

**Fig. 1:**
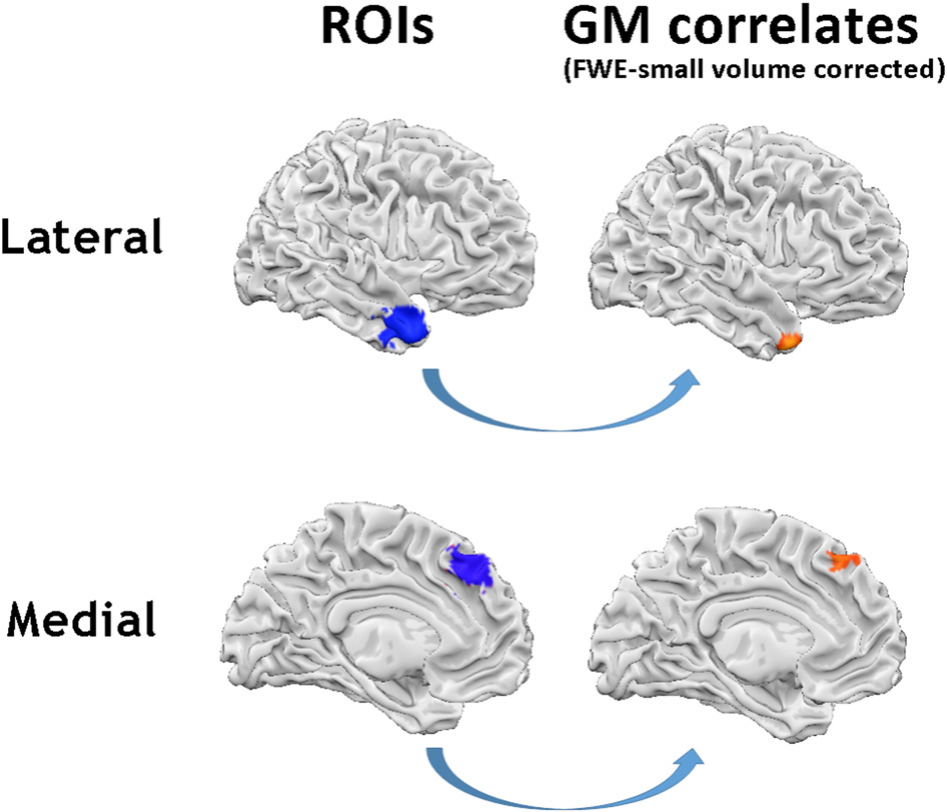
Regions showing negative associations between grey matter volume and urgency, after controlling for age and gender (n = 152) The left-most images show the regions of interest (10 mm diameter spheres set a priori based on previous studies, as detailed in the methods) with the dorsomedial prefrontal cortex sphere at the bottom, and right anterior temporal pole sphere at the top. The images on the right (indicated by arrows) show the grey matter regions negatively associated with urgency scores within these ROIs (p < 0.05 FWE small volume corrected).

**Fig. 2:**
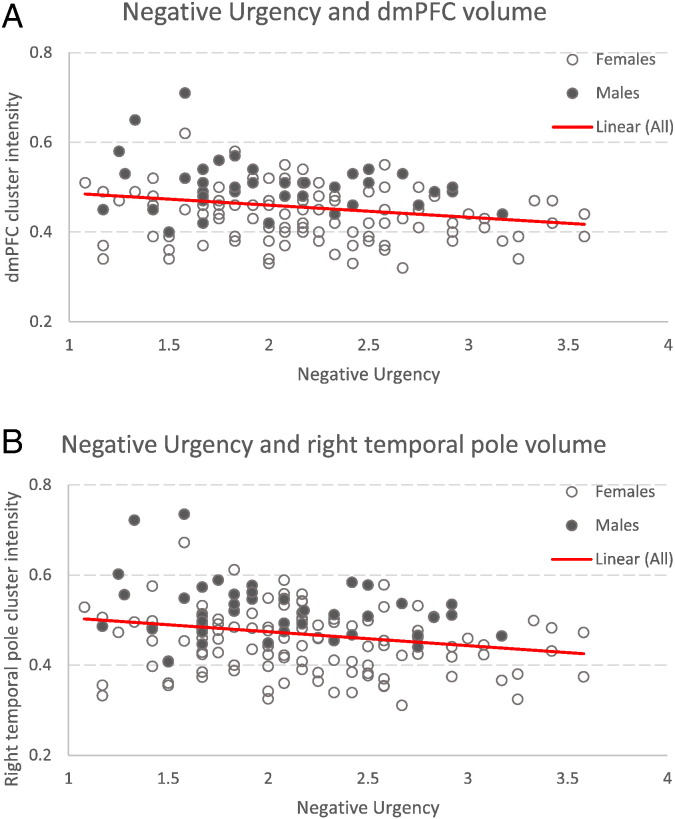
Scatter plots showing the negative association between urgency scores and grey matter volumes within the dorsomedial prefrontal cluster (panel A, top), and the negative association between urgency scores and grey matter volumes within the right temporal pole (panel B, bottom). Associations are shown after correcting for age, gender and using total intracranial volume for global proportional scaling. Correlations are shown for visualisation purposes only.

As predicted, negative associations between urgency scores and grey matter volumes were found in regions of the dorsomedial prefrontal cortex (dmPFC) (MNI coordinates x, y, z = − 4, 33, 48, t = 3.67, cluster extent = 194 mm^3^) and in the right temporal pole (x, y, z = 26, 18, − 38, t = 4.32, cluster extent = 1956 mm^3^), such that higher levels of negative urgency were associated with lower GM volumes in these regions (FWE corrected for multiple comparisons using Gaussian random field theory, small volume corrected) (Fig. 1). There was no evidence for significant gender differences in the relation between regional grey matter volumes and negative urgency in our a priori ROIs (p < 0.001, uncorrected) (Fig. 2).

No positive associations were observed between negative urgency and grey matter volumes in either of the ROIs.

Both the relationship between negative urgency and dmPFC volume and the relationship between negative urgency and right anterior temporal pole volume remained significant after controlling for the other UPPS traits. The same associations with negative urgency were seen within the dmPFC when controlling for levels of neuroticism in the subset of individuals who had completed the BFI-44 in addition to the UPPS (FWE, small volume corrected) (MNI coordinates x, y, z = − 4, 34, 46; t = 3.71, cluster extent = 246 mm^3^).

### Whole brain analyses

**Fig. 3:**
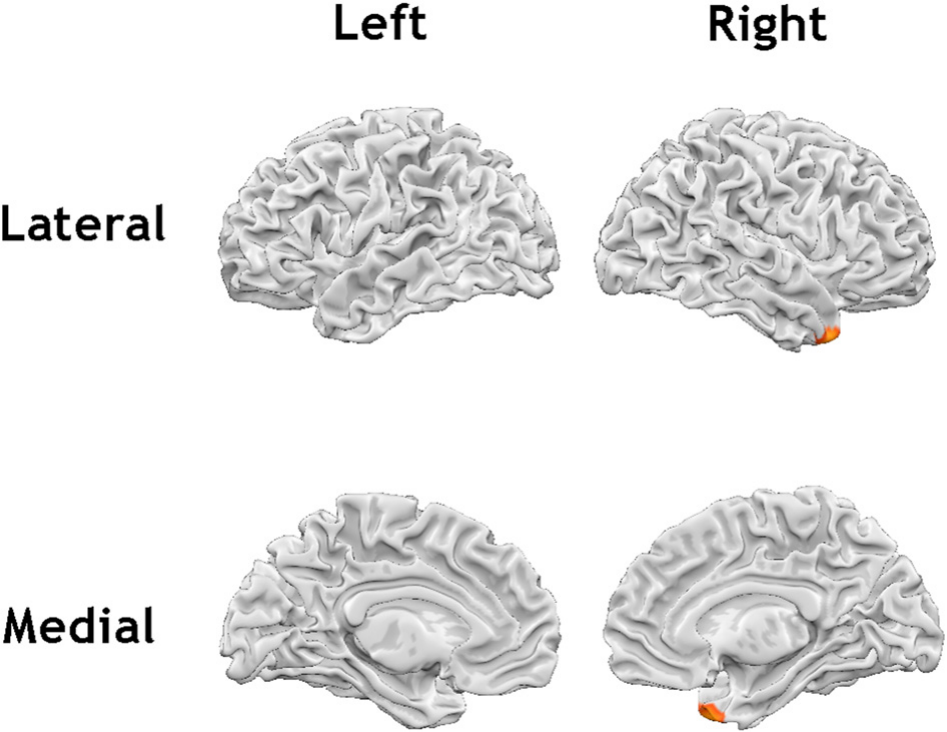
Regions showing a negative association between trait urgency and grey matter volume in the whole brain analysis (p < 0.001, cluster extent corrected for multiple comparisons [k = 456]).

The whole brain analysis revealed a significant negative association between urgency and grey matter volumes in the right temporal pole (x, y, z = 26, 20, − 39, t = 4.4, cluster extent = 1874 mm^3^), after controlling for age and gender (Fig. 3). This was also demonstrated in the reduced subset of 139 participants (x, y, z = 26, 18, − 39, t = 4.32, cluster extent = 1956 mm^3^). The relationship between negative urgency and grey matter volume in the right temporal pole remained significant after additionally controlling for the other UPPS traits (x, y, z = 26, 18, − 38, t = 4.15, cluster extent = 1839 mm^3^).

**Fig. 4:**
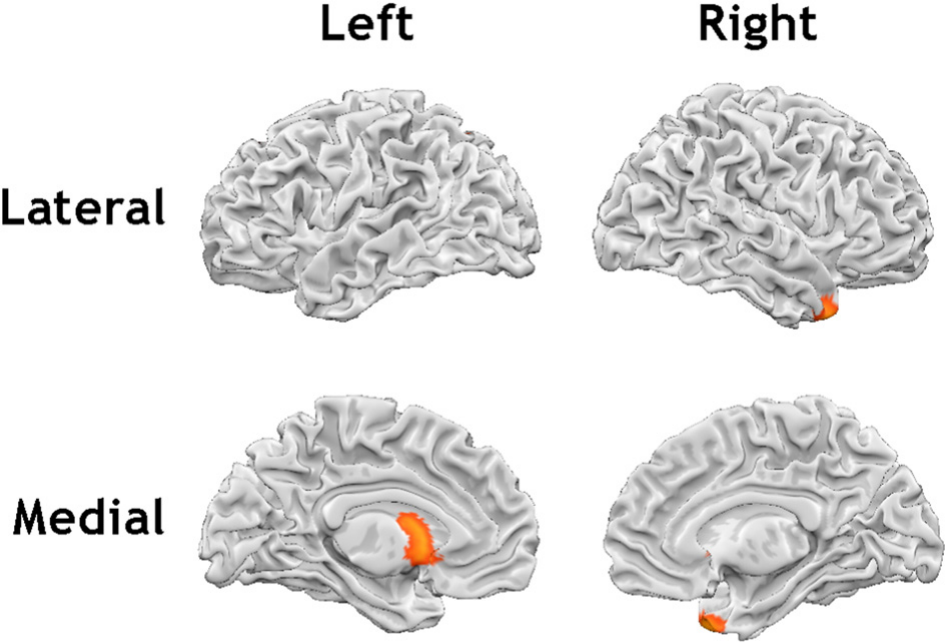
Regions showing negative associations between grey matter volume and urgency, after controlling for levels of neuroticism, in addition to age and gender (n = 139) (p < 0.001, cluster extent corrected for multiple comparisons [k = 456]).

We then examined the relationship between negative urgency and grey matter volume after also controlling for levels of neuroticism in the reduced cohort of 139 participants. This revealed an additional region demonstrating a negative associations between urgency and grey matter volumes in the left ventral striatum (including nucleus accumbens) (x, y, z = − 6, 10, − 2, t = 4.03, cluster extent = 1393 mm^3^) (Fig. 4).

## Discussion

In this study we provide new evidence on the unique brain structure correlates of inter-individual variation in dispositions towards rash, negative emotion-linked impulsivity (negative urgency) in a sample of healthy volunteers. In line with our a priori predictions, we found significant negative associations between trait urgency and grey matter volume in regions of the dmPFC, and in the right anterior temporal pole, such that individuals reporting higher levels of negative urgency (controlling for other UPPS impulsivity traits, age, and gender) showed smaller grey matter volumes in these brain regions. While some previous studies have linked variation in dmPFC volumes or cortical thickness to broad personality measures of impulsivity (Schilling et al., 2012), ours is the first to specifically link variation in dmPFC volume to variation in one specific form of impulsivity, negative urgency, while controlling for levels of other impulsigenic traits. These brain–behaviour associations were also observed after accounting for levels of negative emotionality per se (trait neuroticism), indicating the unique association with negative urgency. Further, when controlling for negative emotionality, higher levels of negative urgency were associated with relatively less regional grey matter in the left ventral striatum, including the nucleus accumbens, a finding that mirrors a recent VBM study of the brain–structure correlates of inter-individual variation in impulsivity in rats (Caprioli et al., 2014).

Amongst the traits delineated in the UPPS model (Whiteside and Lynam, 2001), negative urgency is a uniquely important personality contributor to psychopathology, in particular for the broad spectrum of so-called externalizing or disinhibitory disorders (including various forms of addictive and aggressive disorders) (Birkley and Smith, 2011; Settles et al., 2012). It is therefore notable that lower levels of grey matter volume in the dmPFC, linked here to higher levels of negative urgency, are also seen in a number of different clinical externalizing disorders, including cocaine dependence (Moreno-Lopez et al., 2012; Ide et al., 2014), methamphetamine dependence (Schwartz et al., 2010), alcoholism (Grodin et al., 2013), attention-deficit/hyperactivity disorder (Almeida et al., 2010), and conduct disorder (Fairchild et al., 2011). Lower dmPFC grey matter volumes have also been seen in individuals with suicidality (Giakoumatos et al., 2013) and in those with borderline personality disorder (Rossi et al., 2013), where rash, negative emotion-driven impulsivity is a key feature, and which appear, in part, to be linked to the externalizing spectrum of disorders (Eaton et al., 2011). Thus, structural brain imaging studies suggest a potential shared (“trans-diagnostic”) pattern of relatively low grey matter volume in the dmPFC across several different externalizing disorders that are associated with high levels of rash impulsivity. Furthermore, lower medial frontal grey matter volumes are also seen in individuals at familial high risk for externalizing disorders, including alcohol dependence (Benegal et al., 2007). This latter finding is mirrored in the present study, in that healthy individuals show a continuous relationship between local grey matter volume in the dmPFC and levels of a personality trait (negative urgency) thought to represent the personality “core” of the externalizing spectrum of disorders (Clark, 2005). Our findings are thus consistent with the idea that negative urgency and externalizing have shared neurobiological underpinnings and we suggest that negative urgency might represent an etiologically-relevant personality ‘endophenotype’ for the externalizing disorders, helping in part to explain their systematic co-occurrence (Clark, 2005; Robbins et al., 2012).

Recent research suggests that negative urgency may be of even broader significance to understanding risk for emotional disorders — with negative urgency acting as a prospective predictor of depression, as well as to risk for externalizing disorders (Smith et al., 2013; Johnson et al., 2013). It is notable then, that both unipolar and bipolar depression have also been linked to lower grey matter volume in dmPFC (Ambrosi et al., 2013; Amico et al., 2011; Bora et al., 2012; Brooks et al., 2009), as has schizophrenia (Fornito et al., 2009). Furthermore, Caspi et al. (2013), by examining patterns of co-occurrence of major forms of psychopathology (internalizing, externalizing and thought disorders) in the longitudinal Dunedin birth cohort study, found evidence for a general liability to develop any and all forms of common psychopathology (a so-called ‘p factor’). Higher scores on this factor were associated with the personality traits of (low) conscientiousness, (low) agreeableness and (high) neuroticism — a pattern strikingly similar to that of negative urgency (Settles et al., 2012). Thus, we suggest that negative urgency is relevant to the broad co-occurrence between both externalizing and internalizing disorders, and their overlapping neurobiological substrates.

In addition to a relation with relatively smaller dmPFC grey matter volume, both the ROI-based and whole brain analysis revealed an association between lower grey matter volume in the right temporal pole and increased levels of negative urgency. Lower grey matter volume or thickness in the temporal pole has previously been reported in individuals with attention deficit/ hyperactivity disorder (Fernandez-Jaen et al., 2014), cocaine dependence (Albein-Urios et al., 2013) and in those with borderline and antisocial personality disorders (Bertsch et al., 2013; Boen et al., 2015). In addition, right temporal polar atrophy is observed in frontotemporal lobar dementia, where it has been associated an increase in rash impulsive behaviour (Gorno-Tempini et al., 2004; Chan et al., 2009). Our findings converge with this body of work, by demonstrating associations between inter-individual variation in trait levels of negative urgency and temporal pole grey matter volumes in healthy participants in the absence of either neurotoxic drug effects or neurodegeneration.

Theories of negative urgency conceptualize the trait in terms of inter-individual differences in emotion regulation (Carver et al., 2008), emotion-related decision-making and/or control over actions in the face of intense emotion (Cyders and Smith, 2008). Notably then, the dmPFC has been implicated in various aspects of emotion regulation (Etkin et al., 2011), including the regulation of intense negative emotions (Silvers et al., 2014b) and a recent meta-analysis of fMRI studies of emotion regulation found consistent activation in a region of dmPFC strikingly overlapping with the region in which smaller volumes were linked to higher levels of affect-driven impulsivity in our study (Buhle et al., 2013). The dmPFC is also implicated in the regulation of stress-induced craving (Seo et al., 2011). Furthermore, smaller grey matter volumes in dmPFC have been linked to higher levels of other personality traits aligned with negative urgency, including rumination and neuroticism (DeYoung et al., 2010; Kuhn et al., 2012). Focal lesions of dmPFC impair emotion regulation (Falquez et al., 2014) and also increase risky decision-making on the Iowa Gambling Task (Manes et al., 2002), suggesting a broad role for dmPFC in emotion regulation and emotion-related decision-making. Perhaps most strikingly, Coombes et al. (2012) using fMRI found that dmPFC was involved in preventing emotional states interfering with on-going actions. Recently, variation in trait urgency has been linked to variation in serotonergic functioning (Carver et al., 2011) and so it is of note that dmPFC has selective projections to the serotonergic raphe nucleus (Arnsten and Goldman-Rakic, 1984) and is thus capable of directly influencing serotonergic function.

The role of the dmPFC in emotion regulation and decision making appears to be part of a broader role for this region in higher-level emotional appraisal (Etkin et al., 2011). The dmPFC forms part of the so-called “default mode network” and show increased activity when individuals have to attend to and label or evaluate their own emotional states (Gusnard et al., 2001; Ochsner et al., 2004; Doerig et al., 2013) or retrieve emotional memories (Bado et al., 2014). Further, individuals with depression show reduced activation in dmPFC both when appraising their emotions (Sheline et al., 2009) and in situations (e.g. frustration) when they must regulate their emotions (Schiller et al., 2013) to inhibit rash actions. The temporal pole, where smaller regional GM volumes were also associated with increased levels of negative urgency, is anatomically connected to the dmPFC (Saleem et al., 2008) and also plays a role in emotion regulation and higher-level appraisal of one's emotions. For example, the right temporal pole is activated both during emotion regulation (Ochsner et al., 2002) and when individuals have to verbally label their emotions (Lane et al., 1997). Right temporal pole damage, resulting from ischaemic stroke, anterior temporal lobectomy or frontotemporal dementia, is linked to impaired socio-affective processing including alexithymia (the inability to identify and describe emotions) (Olson et al., 2007). This link between negative urgency, alexithymia and the temporal pole is interesting given that negative urgency is a mediator of the relationship between alexithymia and rash, impulsive behaviours (Shishido et al., 2013). Relatively smaller right temporal polar volumes in individuals with high levels of negative urgency might relate to less effective higher-level cognitive processes relevant to appraising one's emotions and regulating one's emotions (Tavares et al., 2011).

We did not find a significant association between urgency and grey matter volume of other regions linked to emotion regulation, in particular the ventromedial prefrontal cortex (Cyders and Smith, 2008). A recent study of individuals with schizophrenia found that high levels of urgency were associated with lower cortical thickness in the medial, particularly ventromedial, prefrontal cortex (Hoptman et al., 2014). It is possible, however, that the finding in schizophrenia reflects at least in part the effects of antipsychotic medication and/or high levels of substance misuse, which have been shown in animal models to influence MRI measures of cortical thickness (Wheeler et al., 2013; Vernon et al., 2014). Furthermore, a recent meta-analysis (Buhle et al., 2013) failed to find an association between emotion regulation and activity of ventromedial prefrontal cortex, which may play a broader role in emotion generation as well as emotion regulation. Hoptman et al. (2014) did, however, find that high levels of urgency were associated with reduced resting state connectivity of the dorsomedial prefrontal cortex, suggesting some similarities with the current findings.

The negative association between trait urgency and grey matter volume in the left ventral striatum, after controlling for levels of neuroticism is of particular note. Smaller ventral striatal volumes have been reported in a number of clinical externalizing disorders associated with markedly high levels of negative urgency, including alcoholism (Makris et al., 2008) and nicotine addiction (Das et al., 2012). In rats, lesions to the ventral striatum (nucleus accumbens core) increase ‘waiting’ impulsivity, as indexed by an increase in premature responding in a 5-choice serial reaction time task (5-CSRTT) (Christakou et al., 2004) or a reduced ability to delay gratification (Cardinal et al., 2001). In humans, inter-individual variation in negative urgency has been related to the ability to delay gratification (Tsukayama et al., 2012). Further, low levels of ventral striatal dopamine d2 receptor binding are associated both with higher levels of negative urgency in humans (Clark et al., 2012) and with ‘waiting’ impulsivity in rats (Dalley et al., 2007). Most strikingly, a recent VBM study found that rats classified as high on ‘waiting’ impulsivity, as indexed by high-levels of premature responding on the 5-CSRTT, have smaller grey matter volumes in left accumbens relative to low-impulsive rats (Caprioli et al., 2014). This reduced volume was associated with corresponding reductions in levels of gamma-aminobutyric acid (GABA) gene expression in this region. In humans, we have previously reported that lower levels of prefrontal GABA (measured using MR-spectroscopy) are related to higher negative urgency scores (Boy et al., 2011). Our findings thus point to potential similarities between the neurobiology underpinning the trait of negative urgency in humans, and that underpinning specific forms of impulsivity (‘waiting’ impulsivity) in animal models.

There are some limitations that should be considered when interpreting our results. First, as with all cross-sectional studies, there is no indication as to whether our correlations imply causal relationships. There are genetic influences on both negative urgency (Villafuerte et al., 2012) and grey matter volumes within the dmPFC (e.g. Hulshoff Pol et al., 2006). Furthermore, genetic influences partly mediate the relationship between stress reactivity (indexed by salivary cortisol levels) and dmPFC cortical thickness (Kremen et al., 2010), suggesting that genetic factors resulting in relatively reduced functioning of dmPFC and temporal polar regions could result in higher levels of negative urgency. The similarities between our findings and those seen in animal models of impulsivity (Caprioli et al., 2014) would also be consistent with this account. At the same time, given that high levels of negative urgency may exacerbate stress exposure (Liu and Kleiman, 2012), grey matter volumes may be influenced by environmental influences, including stress, that could, over time, impact on regional grey matter volumes (Ansell et al., 2012; van Harmelen et al., 2010). Further, there is evidence to suggest that grey matter volumes are plastic, with increases following the learning of new skills (e.g. Woollett and Maguire, 2011), which could include emotion regulation skills. Collectively, these findings suggest that smaller grey matter volumes in those with high levels of negative urgency likely reflect a complex interplay between genetic and experience-dependent influences (see also Carver et al., 2011) that should be addressed in future longitudinal, genetically-informed studies.

Second, the cellular basis of the factors underlying VBM findings associated with structural MRI are unknown, as is the relationship between brain structure and brain activity as measured by functional MRI (see Kanai and Rees, 2011). Additional studies relating VBM findings to underlying structural and molecular variation in animal models of impulsivity (e.g. Caprioli et al., 2014), will facilitate further understanding of the neurobiology of this key personality trait.

Finally, our sample was not evenly matched for sex, with approximately twice as many females as males. Previous research has found that relations between negative urgency and risk outcomes do not differ by sex (Cyders, 2013), but there is some evidence for sex differences in the neural correlates of emotion regulation (e.g. McRae et al., 2008). While we did not see any significant differences between males and females in the relationship between grey matter volumes and negative urgency scores in our study, we may have been underpowered to detect relatively subtle differences. Future large scale-studies could test potential sex differences in the neural substrates of negative urgency and other impulsigenic traits.

## Conclusions

This study examined the brain structure correlates of rash, negative emotion-based impulsivity, demonstrating that relatively smaller grey matter volumes in the dmPFC, temporal pole and ventral striatum are associated with higher levels of negative urgency. The regional associations we found point to the importance of understanding the contributions of variation in both subcortical structures that likely mediate the overt behavioural manifestations of impulsivity, and within cortical regions that mediate higher-level cognitive processes, such as self-reflective emotional appraisal, to inter-individual variation in impulsivity, the latter of which has until recently been relatively neglected, particularly in animal models of impulsivity. Illuminating the neurobiological variation underpinning inter-individual variation in negative urgency can potentially offer novel insight into factors promoting resilience to problematic risky behaviours and diverse forms of emotional disorder.

## Figures and Tables

**Table 1:** Mean (standard deviation) scores for UPPS impulsivity trait scales and for neuroticism (BFI-44) scores for the whole sample and for females and males separately. Values for each scale are given as mean scores for contributing items. Values in square brackets are those from the participants who completed both the UPPS and the BFI-neuroticism scale. As the number of females remained the same, this information is not repeated. The UPPS measures were scored from a possible 1–4, whereas the neuroticism measure was scored from a possible 1–5. Higher scores reflect higher levels of impulsivity or neuroticism, respectively.

	Total	Females	Males
N	152 [139]	109 [109]	43 [30]
Age	23.6 (5.4) [23.2 (5.3)]	22.0 (3.9)	27.4 (6.3) [27.7 (7.0)]
Urgency	2.1 (0.6) [2.1 (0.6)]	2.2 (0.6)	2.0 (0.5) [2.1 (0.5)]
(Lack of) perseveration	2.1 (0.7) [2.1 (0.7)]	2.1 (0.7)	2.0 (0.5) [2.1 (0.5)]
(Lack of) planning	2.1 (0.5) [2.1 (0.5)]	2.2 (0.5)	1.9 (0.4) [1.9 (0.5)]
Sensation-seeking	2.8 (0.6) [2.8 (0.6)]	2.8 (0.6)	3.0 (0.6) [3.1 (0.6)]
Neuroticism	2.8 (0.8)	3.0 (0.8)	2.4 (0.7)
